# Cognitive screening among acute respiratory failure survivors: a cross-sectional evaluation of the Mini-Mental State Examination

**DOI:** 10.1186/s13054-015-0934-5

**Published:** 2015-05-05

**Authors:** Elizabeth R Pfoh, Kitty S Chan, Victor D Dinglas, Timothy D Girard, James C Jackson, Peter E Morris, Catherine L Hough, Pedro A Mendez-Tellez, E Wesley Ely, Minxuan Huang, Dale M Needham, Ramona O Hopkins, with the NIH NHLBI ARDS Network

**Affiliations:** Department of Health Policy and Management, Johns Hopkins Bloomberg School of Public Health, 615 N. Wolfe Street, Baltimore, MD 21205 USA; Outcomes after Critical Illness and Surgery Group, Johns Hopkins University, 1830 E Monument Street, Baltimore, MD 21205 USA; Division of Pulmonary and Critical Care Medicine, Johns Hopkins School of Medicine, 1830 E Monument Street, Baltimore, MD 21205 USA; Division of Allergy, Pulmonary, and Critical Care Medicine, Department of Medicine, Vanderbilt University School of Medicine, D-3100, Medical Center North, Nashville, TN 37232 USA; Center for Health Services Research, Department of Medicine, Vanderbilt University School of Medicine, 2215 Garland Ave, Nashville, TN 37232 USA; Center for Quality of Aging, Department of Medicine, Vanderbilt University School of Medicine, 2215 Garland Ave, Nashville, TN 37232 USA; Geriatric Research, Education and Clinical Center Service, Department of Veterans Affairs Medical Center, Tennessee Valley Healthcare System, 1310 24th Ave. S, Nashville, TN 37212 USA; Section on Pulmonary, Critical Care, Allergy and Immunologic Diseases, School of Medicine, Wake Forest University, Winston-Salem, NC 27157 USA; Division of Pulmonary and Critical Care Medicine, Harborview Medical Center, University of Washington, Campus Box 356522, Seattle, WA 98195 USA; Department of Anesthesiology and Critical Care Medicine, School of Medicine, Johns Hopkins University, 600 North Wolfe Street, Baltimore, MD 21287 USA; Department of Physical Medicine and Rehabilitation, Johns Hopkins School of Medicine, 1830 E Monument Street, Baltimore, MD 21205 USA; Division of Pulmonary and Critical Care Medicine, Intermountain Medical Center, 5121 Cottonwood Street, Murray, UT 84107 USA; Psychology Department and Neuroscience Center, Brigham Young University, 1022 SWKT, Provo, UT 84602 USA; Center for Humanizing Critical Care, Intermountain Health Care, 5121 South Cottonwood Street, Murray, Utah 84157 USA

## Abstract

**Introduction:**

The Mini-Mental State Examination (MMSE) is a common cognitive screening test, but its utility in identifying impairments in survivors of acute respiratory failure is unclear. The purpose of this study was to evaluate MMSE performance versus a concurrently administered detailed neuropsychological test battery in survivors of acute respiratory failure.

**Methods:**

This cross-sectional analysis used data from the ARDSNet Long Term Outcomes Study (ALTOS) and Awakening and Breathing Controlled Trial (ABC). Participants were 242 survivors of acute respiratory failure. The MMSE and detailed neuropsychological tests were administered at 6 and 12 months post-hospital discharge for the ALTOS study, and at hospital discharge, 3 and 12 months for the ABC study. Overall cognitive impairment identified by the MMSE (score <24) was compared to impairments identified by the neuropsychological tests. We also matched orientation, registration, attention, memory and language domains on the MMSE to the corresponding neuropsychological test. Pairwise correlations, sensitivity, specificity, positive and negative predictive values, and agreement were assessed.

**Results:**

Agreement between MMSE and neuropsychological tests for overall cognitive impairment was fair (42 to 80%). Specificity was excellent (≥93%), but sensitivity was poor (19 to 37%). Correlations between MMSE domains and corresponding neuropsychological tests were weak to moderate (6 months: r = 0.11 to 0.28; 12 months: r = 0.09 to 0.34). The highest correlation between the MMSE and neuropsychological domains was for attention at 6 months (r = 0.28) and language at 12 months (r = 0.34).

**Conclusions:**

In acute respiratory failure survivors, the MMSE has poor sensitivity in detecting cognitive impairment compared with concurrently administered detailed neuropsychological tests. MMSE results in this population should be interpreted with caution.

## Introduction

Many survivors of acute respiratory failure experience long-term cognitive impairments across a variety of cognitive domains, including attention, memory, mental processing speed and executive function [1-6]. To evaluate such impairments, comprehensive neuropsychological test batteries are commonly used [7-11]. However, such batteries require trained personnel, expensive licensing fees and several hours per patient for test administration and scoring. Hence, valid cognitive screening tests that are inexpensive, brief and easy to administer would be invaluable in identifying which survivors may develop long-term cognitive impairment [2]. Furthermore, cognitive screening tests may aid in understanding risk factors and trajectories of cognitive impairments [5,12], and may facilitate the development and evaluation of targeted interventions to address cognitive impairments [13,14].

The Mini-Mental State Examination (MMSE) is a commonly used screening test for cognitive impairment in both clinical practice [15,16], and research [17-24]. Among older adults, the MMSE is the most widely used cognitive screening test, with a pooled sensitivity of 88% and specificity of 86% [15]. Moreover, the MMSE has good performance in identifying mild cognitive impairment in older adults [14], identifying subtypes of mild cognitive impairment [25] and predicting cognitive impairment in patients with post-operative delirium [26].

As a result of these favorable performance characteristics, the MMSE has been commonly used to assess cognitive outcomes in critical care populations [17-22,24]. However, the performance of the MMSE has not been specifically evaluated in acute respiratory failure survivors. Understanding whether the MMSE can accurately screen for cognitive impairment in this patient population would provide important new insights. Hence, the objective of this study is to assess whether the MMSE can detect cognitive impairment, as assessed by a concurrently administered, detailed neuropsychological test battery, in survivors of acute respiratory failure. Additionally, this study explored whether the timing of patient follow-up assessment after hospital discharge or patient characteristics influenced the relationship between the MMSE and the neuropsychological test battery.

## Methods

### Participants

We conducted a cross-sectional secondary analysis of data from two prospective studies of acute respiratory failure patients requiring mechanical ventilation in an ICU. The data from the first study came from the National Institutes of Health-funded ARDS Network Long Term Outcomes Study (ALTOS), which evaluated 6 and 12 month outcomes from patients enrolled in multi-site randomized trials conducted by the ARDS Network from July 2008 to May 2012 [19,27-30]. The patients from these trials had similar eligibility criteria and were enrolled from the same study sites during a similar time frame; as such the data were pooled for this study. Patients in the ALTOS study who completed the MMSE and a concurrent neuropsychological test battery at either 6- or 12-month follow-up (N = 200) were included in the current study.

To increase the generalizability of this evaluation, data from a second study were evaluated. The Awaken and Breathing Controlled (ABC) study evaluated long-term neurocognitive outcomes in patients enrolled at a single hospital in Nashville, Tennessee, between October 2003 and March 2006 [31]. Patients from a sub-study of the ABC study patients were included here [31]. The following baseline and ICU data were obtained: age, gender, race, years of education, employment status, Charlson comorbidity index [32] and psychiatric comorbidity (as described further below), severity of illness, mechanical ventilation duration, and ICU and hospital length of stay.

### ALTOS Study

In the ALTOS study, patients were administered the MMSE and a standardized neuropsychological test battery at 6- and 12-month follow-up [27]. The MMSE was administered via telephone using a validated 26-item version [33], while the neuropsychological battery was administered in person by research assistants who underwent detailed training and quality assurance evaluations by RH. The telephone version of the MMSE differs from the in-person MMSE in that it excludes the following items: 1) A question about orientation to place, which asks ‘What floor of the building are we on?’; 2) A question related to following commands which asks the patient to ‘close your eyes’; 3) A question related to simple sentence construction that asks the patient to ‘write a sentence’ 4) A question related to visuospatial construction that asks the patient to ‘copy overlapping pentagons’. Additionally, the telephone version uses only one naming item, as opposed to three items in the standard MMSE [33]. The MMSE telephone score was converted to the standard 30-point scale of the standard MMSE using previously published methods [33]. The MMSE assesses overall cognitive impairment based on five cognitive domains: 1) orientation (orientation to time and place), 2) registration (registration of three words), 3) attention (backward spelling of ‘WORLD’), 4) memory (recall of three words) and 5) language (naming, repetition and following a three-stage command) [34].

Similar cognitive domains were identified from the neuropsychological test battery, which is made up of widely used neuropsychological tests with known reliability and validity [35-37]. The domains included: 1) orientation from the Neurobehavioral Cognitive Status Examination orientation total score [38]; 2) registration from the Digit Span’s raw forward score from the Wechsler Adult Intelligence Scale-Third Edition [39,40]; 3) attention from the Digit Span’s total age-adjusted score and the raw forward and backward scores from the Wechsler Adult Intelligence Scale-Third Edition [39,40]; 4) memory from the Logical Memory I and Logical Memory II age-adjusted scores from the Wechsler Memory Scale-Third Edition [39,40] and 5) language via the Verbal Fluency test [41]. MMSE scores for each domain were compared to scores for the corresponding neuropsychological test (recognizing there is not necessarily a one-to-one correspondence between the tests in the domains of interest between the MMSE and neuropsychological test battery). We also compared overall cognitive impairment between the MMSE and the neuropsychological test battery.

Presence of baseline psychiatric comorbidity was present if the patient had any of the following (based on medical chart review): current or past excessive alcohol use, illicit drug use or drug rehabilitation, diagnosis or treatment for psychiatric disorder or documented history of depression or anxiety [19,42]. Depression and anxiety were assessed at 6 and 12 months using the Hospital Anxiety and Depression Scale, which provides separate subscale scores for depression and anxiety. Scores of eight or higher indicate probable depression or anxiety [19,43].

### ABC study

In the ABC study, patients were administered the MMSE and a detailed neuropsychological test battery in person at hospital discharge, and at 3- and 12-month follow-up, by a single neuropsychologist [31]. To examine generalizability of the primary ALTOS study analyses, we evaluated the ability of the MMSE to detect overall cognitive impairment compared to a similar neuropsychological test battery in the ABC study [31]. The ABC study test battery included: 1) Digit Span and Digit Symbol from the Wechsler Adult Intelligence Scale-Third Edition [39,40]; 2) the Rey Auditory Verbal Learning Test (RAVLT) [44]; 3) Rey-Osterreith Complex Figure-Copy and Delayed Recall [45]; 3) Trail Making Test Parts A and B [46] and 4) Verbal Fluency test [47]. We also used data from the ABC study to compare the MMSE attention domain with the Digit Span scores [39].

### Statistical analysis

Cross-sectional data analysis compared MMSE scores with neuropsychological test scores at 3, 6 and 12 months. Cognitive impairment was defined for the MMSE as total scores <27 and <24, which represent widely used, highly sensitive and specific cutoff scores for cognitive impairment [17,48]. Sensitivity and specificity were similar when comparing total MMSE scores of <27 and <24 versus neuropsychological test scores; therefore, we only report the more conservative cutoff of <24. For the neuropsychological test batteries, cognitive impairment was conservatively defined, as done in prior studies [19,27,31], as having at least one cognitive test ≥2 standard deviations below population norms (bottom 2.5%), or at least two tests ≥1.5 standard deviations below population norms (bottom 6.7% for both tests). The ability of the MMSE to detect cognitive impairment was evaluated using sensitivity, specificity, positive and negative predictive values, and agreement. The area under the receiver operating characteristics curves was also used to examine the ability of the total MMSE score to discriminate between survivors with and without cognitive impairment, as defined by the neuropsychological test battery [19,27-31].

Pearson correlations were used to examine the relationship between MMSE cognitive domains and the corresponding cognitive domains from the neuropsychological test results. We examined whether these relationships were affected by patient characteristics. Correlations were calculated within relevant patient subgroups defined by age (dichotomized at the mean age of 50 years), gender, education level (dichotomized at the mean of 12 years, that is, high school graduation), depression and anxiety status at 6 and 12 months using the Hospital Anxiety and Depression Scale [43], and pre-existing psychiatric comorbidity prior to acute respiratory failure. Statistical significance was defined as *P* ≤0.05 and statistical analyses were completed using STATA 12.0 (StataCorp, College Station, TX, USA). This study was conducted in accordance with the amended Declaration of Helsinki. All studies obtained informed consent from participants, and were approved by relevant institutional review boards (ALTOS: Johns Hopkins School of Medicine Institutional Review Board-5 approval number: NA_00013113; ABC: Vanderbilt University and Saint Thomas Hospital Institutional Review Board number: 030803).

## Results

In the ALTOS study, of the 200 eligible patients, 181 and 174 patients completed both the MMSE and the neuropsychological tests at 6 and 12 months, respectively. In the ABC study, 61, 69 and 47 participants completed both the MMSE and neuropsychological tests at hospital discharge, 3 months and 12 months, respectively. Respondents in both studies were predominately white, middle-aged and male, with a mean of approximately 12 years of education (Table 1). Pre-ICU psychiatric comorbidity was common, with a prevalence of 42% in the ALTOS study and 74% in the ABC study. All patients were mechanically ventilated, and the mean ICU length of stay was 14 and 10 days in the ALTOS and ABC studies, respectively. For the ALTOS study, 12% and 10% of patients had MMSE scores <24 at 6 and 12 months, respectively; while 38% and 25% of patients had cognitive impairments based on the neuropsychological test scores at 6 and 12 months. In the ABC study, the percentage of patients with an MMSE score <24 was 33%, 17% and 13% at discharge, 3 months and 12 months respectively; while 90%, 79% and 71% had cognitive impairments based on the neuropsychological test scores at these same time points, respectively.

**Table 1:** **Patient characteristics**

**Characteristic**	**ALTOS (n = 181)**	**ABC trial (n = 61)**
Age, mean (SD) years	49	58 (16)
Male, n (%)	90 (50)	38 (62)
Education years, mean (SD)	13 (3)	12 (3)
Employed, n (%)	93 (51)	NA
Charlson Comorbidity Index, mean (SD)	1.2 (1.7)	NA
Psychiatric comorbidity, n (%)	76 (42)	45 (74)
Severity of illness score, mean (SD)	APACHE III: 85 (25)	APACHE II: 28 (8)
Mechanical ventilation duration, mean (SD) days	11 (10)	6 (6)
ICU length of stay, mean (SD) days	14 (11)	10 (8)
Hospital length of stay, mean (SD) days	22 (15)	14 (8)

ICU, Intensive Care Unit; SD, standard deviation; n, number.

### Comparing overall cognitive impairment

**Figure 1:**
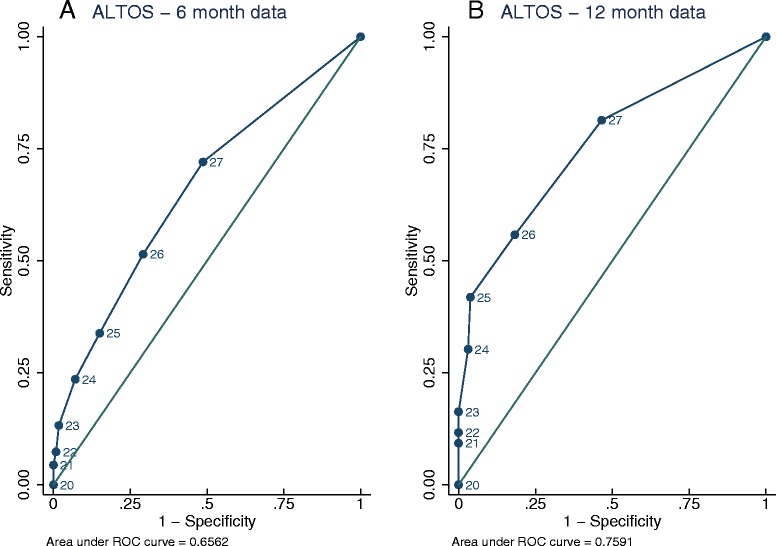
Receiver operating characteristics curve for the detection of overall cognitive impairment using the MMSE versus a detailed neuropsychological test battery. **A**. ALTOS study 6-month data and **B**. ALTOS study 12-month data. ALTOS, ARDS Network Long Term Outcomes Study; MMSE, Mini-Mental State Examination.

For the ALTOS study, at 6 and 12 months, overall agreement was 67% and 80%, respectively. Kappa indicated poor concordance after accounting for chance agreement (0.19 and 0.34). At 6 and 12 months, the specificity of the MMSE was excellent (93% and 97%, respectively); however, sensitivity was poor (24% and 30%, respectively), and positive predictive values (67% and 76%, respectively) and negative predictive values (67% and 81%, respectively) were fair to moderate (Table 2). The area under the receiver operating characteristics curve was 0.66 (95% CI: 0.58 to 0.74) at 6 months and 0.76 (95% CI: 0.67 to 0.85) at 12 months, indicating fair discrimination (Figure 1).

**Table 2:** **Overall cognitive impairment at discharge, 3, 6 and 12 months for MMSE versus neuropsychological test battery**

	**Sensitivity**		**Specificity**		**Positive predictive value**		**Negative predictive value**		**Kappa (SD)**	**Overall agreement (%)**
	**%**	**(95% CI)**	**%**	**(95% CI)**	**%**	**(95% CI)**	**%**	**(95% CI)**		
**ALTOS Data**										
**6 Months**										
MMSE <24	24	(14–35)	93	(87–97)	67	(45–84)	67	(59–74)	0.19 (0.06)	67
**12 Months**										
MMSE <24	30	(17–46)	97	(92–99)	76	(50–93)	81	(74–87)	0.34 (0.07)	80
**ABC Data**										
**Discharge**										
MMSE <24	37	(26–49)	100	(59–100)	100	(87–100)	13	(6–26)	0.09 (0.05)	42
**3 Months**										
MMSE <24	22	(12–34)	100	(79–100)	100	(75–100)	25	(15–38)	0.10 (0.05)	38
**12 Months**										
MMSE <24	19	(8–35)	100	(78–100)	100	(59–100)	33	(20–49)	0.12 (0.07)	42

MMSE, mini-mental status examination; SD standard deviation.

Impairment on neuropsychological test battery = any battery test ≤2 SD or 2+ tests ≤ 1.5 SD.

For ALTOS study data*:* At 6 months: N = 181; True positives = 9%; False positives = 4%; True negatives = 58%; False negatives = 29%. At 12 months: N = 174; True positives = 7%; False positives = 2%; True negatives = 73%; False negatives = 17%.

For ABC study data*:* At discharge: N = 78; True positives = 33%; False positives = 0%; True negatives = 9%; False negatives = 58%. At 3 months: N = 76; True positives =17%; False positives = 0%; True negatives = 21%; False negatives = 62%. At 12 months: N = 52; True positives = 13%; False positives = 0%; True negatives = 29%; False negatives = 58%.

For the ABC study data, overall agreement and kappa scores were low (ranging from 38 to 42%, and 0.09 to 0.12, respectively) when assessing concordance between screening positive for cognitive impairment on the MMSE, and having cognitive impairment based on the neuropsychological test scores. Specificity and positive predictive value of the MMSE was excellent, at 100% for all three time points. Sensitivity was poor, ranging from 19 to 37%. Negative predictive values were also poor at all three time points: 13% at discharge, 25% at 3 months and 33% at 12 months (Table 2).

### Comparing specific cognitive domains

Correlations between cognitive domains in the MMSE compared to neuropsychological domains were weak to moderate (6 months: 0.11 to 0.28, Table 3; 12 months: 0.09 to 0.34, Table 4), with the highest correlations observed for attention at 6 months (r = 0.28) and language at 12 months (r = 0.34). There was no consistent influence of patient characteristics (gender, age, education level, concurrent depression and anxiety status, or pre-existing psychiatric comorbidity) on the correlations in any cognitive domain. The majority of correlations in the subgroup analyses were ≤0.30.

**Table 3:** **Correlations of MMSE domain scores with corresponding neuropsychological test by demographic variables at six months**

**MMSE domain**	**Corresponding neuropsychological test**	**All patients**	**Gender**		**Age**		**Education**		**Depression**		**Anxiety**		**Psychiatric**	
									**(Cutoff ≥8)**		**(Cutoff ≥8)**		**Co-morbidity**	
		**(N = 181–183)**	**Male**	**Female**	**<50**	**≥50**	**≤HS**	**>HS**	**No**	**Yes**	**No**	**Yes**	**No**	**Yes**
			**(n = 90)**	**(n = 90–92)**	**(n = 82–84)**	**(n = 99)**	**(n = 92)**	**(n = 88)**	**(n = 115)**	**(n = 65)**	**(n = 107)**	**(n = 74)**	**(n = 90–91)**	**(n = 88–89)**
**Orientation**	**Orientation total**	0.17^a^	0.28^a^	−0.01	0.05	0.24^a^	0.16	−0.08	0.34^a^	0.05	0.10	0.23^a^	0.05	0.19
**Registration**	**DS total score**	0.16^a^	0.15	0.16	0.11	0.20	0.25	0.10	0.23^a^	0.03	0.24^a^	0.08	0.11	0.20
	**DS forward**	0.16^a^	0.23^a^	0.09	0.09	0.21^a^	0.31^a^	0.03	0.25^a^	0.00	0.34^a^	0.01	0.18	0.11
	**DS backward**	0.13	0.02	0.21	0.11	0.13	0.13	0.12	0.19^a^	0.01	0.15	0.10	0.01	0.23
**Attention**	**DS total score**	0.28^a^	0.42^a^	0.03	0.29^a^	0.26^a^	0.32^a^	0.08	0.26^a^	0.27^a^	0.24^a^	0.32^a^	0.26^a^	0.29^a^
	**DS forward**	0.24^a^	0.33^a^	0.02	0.30^a^	0.18^a^	0.25^a^	0.07	0.23^a^	0.23	0.27^a^	0.20	0.26^a^	0.21
	**DS backward**	0.26^a^	0.42^a^	0.00	0.23^a^	0.28^a^	0.32^a^	0.05	0.27^a^	0.22	0.14	0.36^a^	0.20	0.32^a^
**Memory**	**LM I**	0.13	0.07	0.23*	0.12	0.15	0.15	0.06	0.08	0.19	0.21^a^	0.01	0.17	0.12
	**LM II**	0.10	0.00	0.20	0.08	0.15	0.13	0.00	0.04	0.17	0.20^a^	−0.08	0.14	0.09
**Language**	**COWA**	0.16^a^	0.24^a^	0.08	0.04	0.22^a^	0.22^a^	0.10	0.10	0.16	0.10	0.21	0.15	0.18

MMSE, mini-mental status examination; COWA, controlled oral word association; DS, digit span; LM, logical memory, HS, high school.

^a^ *P* ≤0.05.

**Table 4:** **Correlations of MMSE domain scores with corresponding neuropsychological test by demographic variables at 12 months**

**MMSE domain**	**Corresponding neuropsychological test**	**All patients**	**Gender**		**Age**		**Education**		**Depression**		**Anxiety**		**Psychiatric**	
									**(Cutoff ≥8)**		**(Cutoff ≥8)**		**Co-morbidity**	
		**(N = 173–174)**	**Male**	**Female**	**<50**	**≥50**	**≤HS**	**>HS**	**No**	**Yes**	**No**	**Yes**	**No**	**Yes**
			**(n = 83)**	**(n = 90–91)**	**(n = 78)**	**(n = 95–96)**	**(n = 88–89)**	**(n = 83)**	**(n = 117–118)**	**(n = 57–56)**	**(n = 106–107)**	**(n = 67–68)**	**(n = 87–88)**	**(n = 85)**
**Orientation**	**Orientation total**	0.09	0.05	0.14	0.22	0.01	0.09	−0.01	0.13	−0.05	0.05	0.10	0.14	0.06
**Registration**	**DS total score**	0.13	0.15	0.10	0.12	0.15	0.15	0.13	0.02	0.28^a^	0.03	0.28^a^	0.03	0.24^a^
	**DS forward**	0.14	0.13	0.14	0.11	0.16	0.15	0.15	0.01	0.29^a^	0.01	0.29^a^	−0.02	0.30^a^
	**DS backward**	0.13	0.18	0.06	0.11	0.16	0.14	0.13	0.04	0.25	0.04	0.24	0.06	0.21
**Attention**	**DS total score**	0.28^a^	0.34^a^	0.20	0.27^a^	0.28^a^	0.38^a^	−0.01	0.08	0.43^a^	0.23^a^	0.37^a^	0.27^a^	0.29^a^
	**DS forward**	0.26^a^	0.31^a^	0.16	0.33^a^	0.20	0.32^a^	0.02	0.09	0.37^a^	0.22^a^	0.31^a^	0.25^a^	0.27^a^
	**DS backward**	0.28^a^	0.36^a^	0.20	0.20	0.33^a^	0.39^a^	−0.10	0.11	0.42^a^	0.25^a^	0.33^a^	0.29^a^	0.28^a^
**Memory**	**LM I**	0.16^a^	0.23^a^	0.11	0.17	0.16	0.11	0.07	0.19^a^	0.05	0.16	0.14	0.15	0.17
	**LM II**	0.23^a^	0.30^a^	0.18	0.22	0.25^a^	0.18	0.13	0.25^a^	0.15	0.21^a^	0.23	0.20	0.26^a^
**Language**	**COWA**	0.34^a^	0.37^a^	0.34^a^	0.45^a^	0.27^a^	0.34^a^	0.20	0.35^a^	0.25	0.44^a^	0.16	0.29^a^	0.43^a^

MMSE, mini-mental status examination; COWA, controlled oral word association; DS, digit span; LM, logical memory, HS, high school.

^a^ *P* ≤0.05.

Compared to the ALTOS study, the ABC study data had slightly stronger correlations for attention at all time periods (<0.5 versus <0.3). However, in both the ALTOS and ABC studies, there was no consistent influence of gender and age on correlations between MMSE and neuropsychological attention scores (Table 5).

**Table 5:** **Correlations of MMSE domain scores with corresponding neuropsychological test by demographic variables, ABC study data**

**MMSE domain**	**Corresponding neuropsychological test**	**All patients**	**Gender**		**Age**	
			**Male**	**Female**	**<50**	**≥50**
**At Discharge:**		(N = 61)	(n = 38)	(n = 23)	(n = 17)	(n = 44)
Attention	Digit Span total score	0.47^a^	0.55^a^	0.39	0.49^a^	0.46^a^
	Digit Span forward	0.29	0.32	0.31	0.32	0.28
	Digit Span backward	0.45^a^	0.53^a^	0.34	0.63^a^	0.41^a^
**3 Months:**		(n = 68–69)	(n = 37)	(n = 31–32)	(n = 19)	(n = 49–50)
Attention	Digit Span total score	0.34^a^	0.38^a^	0.29	−0.30	0.42^a^
	Digit Span forward	0.20	0.27	0.12	−0.16	0.22
	Digit Span backward	0.39^a^	0.35^a^	0.45^a^	−0.41	0.51^a^
**12 Months:**		(n = 45–47)	(n = 21–22)	(n = 24–25)	(n = 10)	(n = 35–37)
Attention	Digit Span total score	0.47^a^	0.48^a^	0.47^a^	0.62	0.45^a^
	Digit Span forward	0.20	0.31	0.12	0.50	0.17
	Digit Span backward	0.49^a^	0.57^a^	0.33	0.32	0.52^a^

MMSE, mini-mental status examination.

^a^ *P* ≤0.05.

## Discussion

In cross-sectional analyses of two prospective longitudinal studies of acute respiratory failure survivors, we found that the MMSE had fair to moderate agreement, both for overall cognitive impairment and for specific cognitive domains, compared to more detailed neuropsychological tests. These findings were not influenced by patient characteristics or timing of longitudinal follow-up assessment. Indeed, even when accounting for gender, age, education level, concurrent depression and anxiety status, and pre-existing psychiatric comorbidity prior to ICU admission, MMSE domain scores correlations were weak to moderate with neuropsychological test scores.

The ability of the MMSE to discriminate overall cognitive impairment was only fair based on the receiver operating characteristics curve analysis; expressly, the MMSE had low sensitivity. Even using a conservative cutoff score of <24, the MMSE failed to identify a substantial portion of survivors who had cognitive impairment identified on neuropsychological tests, with poor to moderate negative predictive values. Generally, a screening tool should have an area under the ROC curve of at least >0.8 to demonstrate excellent diagnostic accuracy [49], and the MMSE failed to meet this threshold at both 6 and 12 months. These results are similar to previous work in cardiac surgery [50] and bariatric surgery [51] patients. Understanding the limited extent of the MMSE’s agreement with neuropsychological test scores administered concurrently has important implications. Specifically, screening negative on the MMSE for cognitive impairment does not rule out the presence of cognitive impairment for acute respiratory failure survivors at 3, 6 and 12 months.

There is great interest in screening for cognitive impairment in ICU survivors given the frequent and long-lasting cognitive impairments observed [52]. Our study provides additional evidence that the MMSE may not be a valid cognitive screening tool for use in this patient population. Evaluation of other cognitive screening instruments (such as the Montreal Cognitive Assessment) in this population is needed to determine if they will better identify cognitive impairments [53]. Recent work found the Montreal Cognitive Assessment is more sensitive in accurately differentiating mild cognitive impairment from normal cognitive function [54]. Another approach could include augmenting the MMSE with more targeted instruments. For example, adding a measure of executive function to the MMSE can improve the detection of cognitive deficits [55].

The MMSE is characterized by highly selective coverage of cognitive domains and does not evaluate learning, delayed memory, processing speed and executive function, among other cognitive domains. Many of these omitted cognitive domains reflect the frontal-subcortical white matter-mediated impairments known to occur in critical illness, and commonly identified in ICU survivors [4,14,27,31,56]. Hence, their omission in a short screening test is problematic. As we learn more about the granular and specific aspects of cognitive impairment in acute respiratory failure survivors, better cognitive screening tools tailored to this population could be developed.

Our study is among the first to test the validity of the MMSE in acute respiratory failure survivors. Strengths of our study include the ability to examine overall and domain-specific performance of the MMSE compared to a battery of neuropsychological tests administered concurrently. The ALTOS study provided a relatively large multicenter sample, which employed both a screening and neuropsychological test battery, and data from two time points. Inclusion of the ABC study allowed us to test the generalizability of our findings in an independent cohort using similar neuropsychological tests. Our study also has potential limitations. First, we were not able to fully replicate all of the analyses from the ALTOS data using the ABC study data due to the different follow-up time points and neuropsychological tests used. However, the available comparisons (overall cognitive impairment and attention) demonstrated similar results to the ALTOS study, helping increase confidence in the generalizability of our findings, especially since the studies used different neuropsychological tests. Further, participants in the ABC study had a high baseline psychiatric comorbidity, which is higher than the prevalence observed in other ICU cohorts [57,58]. However, while the prevalence of baseline psychiatric comorbidities was different between the two studies (42% for the ALTOS study and 74% for the ABC study), the results of the analyses of the MMSE were similar. Second, despite the relatively large sample size, secondary analyses conducted using binary patient categories had smaller sample sizes and may have reduced power to detect true difference between subgroups. Third, the MMSE and neuropsychological tests were administered using different methods in the ALTOS study (phone versus in-person), potentially contributing to the differences. However, the MMSE is validated for telephone administration, minimizing this concern [34]. Moreover, in-person administration was used for all testing in the ABC study and demonstrated similar results to the ALTOS study. Finally, the MMSE total score is validated to identify cognitive impairment; however, the domain scores have not been validated as standalone measures. Our assessment of the correlations between the MMSE domains and the corresponding neuropsychological tests should be taken within context of overall study findings, and not as a recommendation to only administer specific domains of the MMSE.

## Conclusions

Identifying valid, reliable and feasible screening tools is vital for evaluating long-term cognitive impairments commonly occurring in acute respiratory failure survivors. The MMSE, one of the most commonly used cognitive screening tools, demonstrated only fair agreement and poor sensitivity when compared to concurrently administered neuropsychological test batteries in acute respiratory failure survivors during their first year of recovery. Our findings do not support use of the MMSE as a screening tool for cognitive impairment in this patient population. Evaluation of alternative cognitive screening tests, either alone or in combination with other measures, is needed in acute respiratory failure survivors.

## Key messages

- The Mini-Mental Status Examination (MMSE) is widely used as a screening tool for cognitive impairment in other patient populations, but its ability to detect impairment in survivors of acute respiratory failure is unclear.
- In survivors of acute respiratory failure, the MMSE (compared to a detailed neuropsychological test battery) demonstrated moderate agreement in detecting overall cognitive impairment, with excellent specificity but poor sensitivity.
- The area under the receiver operating characteristics curve indicates the MMSE had only fair discrimination in detecting cognitive impairment at 6 and 12 months.
- Our findings were not influenced by patient characteristics (age, gender or education), psychiatric disorders or timing of longitudinal follow-up assessment.
- MMSE results in survivors of acute respiratory failure should be interpreted with caution.

## Abbreviations

ABC: Awakening and Breathing Controlled Study
ALTOS: ARDSNet Long Term Outcomes Study
ICU: Intensive care unit
MMSE: Mini-Mental State Examination
